# A Review of Immunocryosurgery and a Practical Guide to Its Applications

**DOI:** 10.3390/diseases9040071

**Published:** 2021-10-14

**Authors:** Georgios Gaitanis, Ioannis D. Bassukas

**Affiliations:** 1 Department of Skin and Venereal Diseases, Faculty of Medicine, School of Health Sciences, University of Ioannina, 45110 Ioannina, Greece; ibassuka@uoi.gr; 2 Delc Clinique, 2502 Biel/Bienne, Switzerland

**Keywords:** imiquimod, cryosurgery, immunocryosurgery, basal cell carcinoma, keratinocytic cancers, actinic keratosis, squamous cell carcinoma, keratoacanthoma

## Abstract

Immunocryosurgery is a minimally invasive combinational therapeutic procedure that has been designed, developed, and evaluated in the Dermatology Department of the University of Ioannina from 2004. In a fixed time protocol, this approach combines immune stimulatory therapy with imiquimod and cryosurgery, i.e., cryosurgery is applied during continuous imiquimod treatment. Laboratory findings in tissue and blood level credit the efficacy to the synergy of imiquimod and cryosurgery. The synergy has been established through clinical trials and the excellent feasibility and efficacy demonstrated in clinical practice. Immunocryosurgery has extensive proof of excellent efficacy, comparable to surgery, in the treatment of basal cell carcinoma. It has also been evaluated in cases of Bowen’s disease, keratoacanthoma, Merkel cell carcinoma, *lentigo maligna*, and cutaneous squamous cell carcinoma with or without the addition of adjuvants. The aims of this review are to detail the immunocryosurgery protocol with the addition of daily practice clinical tips, compile data on the mechanism of action of immunocryosurgery, and delineate indications and possible future applications. Most of the available data originate from the treatment of BCC, of all histological types and localizations, and the principles reported mainly reflect on evidence related to the treatment of this common skin cancer.

## 1. Introduction

Immunocryosurgery is a minimally invasive combinational therapeutic procedure that has been designed, developed, and evaluated in the Department of Dermatology in the University of Ioannina Medical School from 2004 [1]. In a fixed time protocol, this approach combines immune stimulatory therapy as is imiquimod with cryosurgery.

The combination of two therapeutic approaches is based on the synergy or at least on the complementarity of the respective therapeutic mechanisms. Thus, during the development period, immunocryosurgery was designed based on general principles of cancer immunotherapy and the theoretical implementation of published data on the anticancer modes of action of the two combined modalities [2]. The postulated combinational activity was subsequently confirmed through the excellent feasibility and efficacy findings recorded during the evaluation of the modality on the following clinical trials phase. Up to now, immunocryosurgery has extensive proof of distinct efficacy, comparable to surgery, in the treatment of basal cell carcinoma (BCC) [3,4,5]. Further indications, mainly based to date on evidence from cases and case-series observations, include Bowen’s disease [6], keratoacanthoma [7], Merkel cell carcinoma [8], *lentigo maligna* [9], and, more recently, cutaneous squamous cell carcinoma (cSCC) [10] with or without the addition of adjuvants. Relevant laboratory findings credit the effectiveness of this approach to the synergy of imiquimod and cryosurgery [2,3,11,12].

The aims of this review are to detail the immunocryosurgery protocol with the addition of practical tips for its successful application in the every-day clinical practice, compile data on the mechanism of action, and to delineate indications and possible future applications. Most of the available data originate from the treatment of BCC, of all histological types and localizations, and the general principles reported herein mainly reflect on evidence and experience related to the treatment of this common skin cancer.

## 2. Action Mechanism of Immunocryosurgery

**Figure 1:**
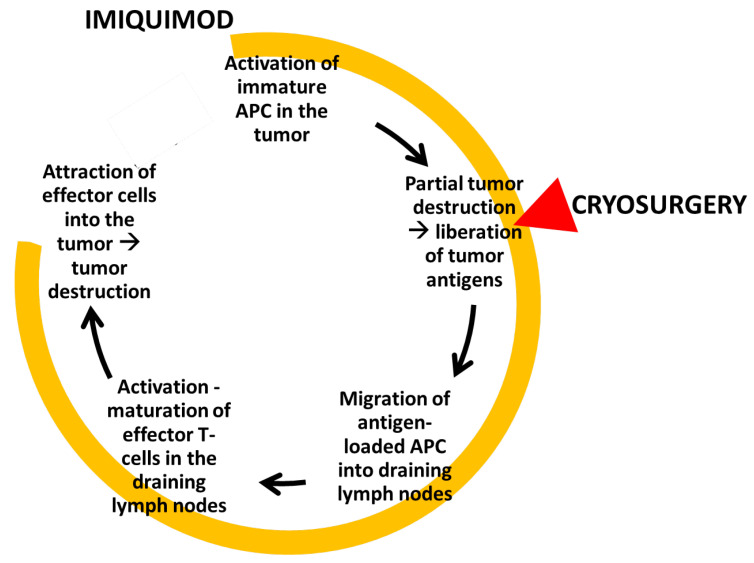
Graphic depicting the cellular events that ensue during a standard immunocryosurgery session: The application of imiquimod attracts immature APCs in the tumor area which uptake the tumor antigens liberated during and after the cryosurgery session. Further application of imiquimod results in the attraction of effector cells that lead to tumor destruction. APC: Antigen-presenting cells.

The combination of imiquimod and cryosurgery has a spectrum of synergistic actions that result in the enhancement of their respective therapeutic action [13,14]. It should be stressed that, although the cryosurgery session is a defining timepoint in the treatment protocol, it cannot be separated from the imiquimod treatment as the high anti-tumor activity results from the combination of a continuous, uninterrupted application of imiquimod and the intercalary cryosurgery session:

- The precryosurgery treatment of the tumor with imiquimod creates a proapoptotic environment within and around the tumor [15] that intensifies the destruction of the tumor cells during the ‘physical’ phase of the upcoming cryosurgery. This is especially important at the periphery of the treated lesion where effective freezing temperatures cannot be achieved without extensive damage to the adjacent healthy tissues.
- Immunocryosurgery may further enhance vascular tumor bed destruction by promoting initial damage of vessel endothelia (e.g., via apoptosis) and by restricting the induction of adequate tumor neovascularization after the cryosurgical insult [16,17].
- Finally, data originating from BCC studies suggest that a cascade of tissue events during immunocryosurgery leads to an efficient antitumor immune response (Figure 1).

Contrary to surgery, which removes the tumor tissue, immunocryosurgery as an ablative therapeutic modality yields tumor destruction in situ, enabling a vivid intervention of immunological mechanism. Tumors have developed diverse mechanisms to defend against host’s immune attack [18]. BCCs, for example, grow within a Th2 cytokine-dominated ‘tumor-protective’ immunological microenvironment [19] which can be partially restored by topical imiquimod [15]. The cornerstone event for this is the in loco activation of antigen-presenting cells (APCs), particularly immature plasmacytoid dendritic cells (pDCs) that form one of the earliest steps in the antineoplastic immune response cascade [20]. Tumor-infiltrating APCs are present in untreated BCCs [19] but are most probably functionally ‘inactivated’, and newly recruited pDCs are essential for the mobilization of the immune response [21]. Naive APCs are attracted by imiquimod into the tumor [17,22]; however, in the clinical practice, this often leads to only partial tumor destruction. The balance between immunogenic cell necrosis and tolerance-promoting apoptosis is crucial for the immunological net result of all tumor-ablative therapies. Probably the proapoptotic mechanisms of the imiquimod prevail over cell necrosis in above cases, and thus limit the immune stimulation and consequently the tumor-destructive inflammatory response. At this point, the role of the induction of massive cell necrosis by the cryosurgical insult seems to be crucial, as through the partial destruction of the tumor tissue a large amount of many different, structurally intact immunogenic tumor antigens are liberated in situ, directly in the pre-conditioned neoplastic microenvironment [23]. The tumor peptides are subsequently picked up by the imiquimod-attracted APCs and transferred into the draining lymph nodes where they are presented to T lymphocytes, inducing their activation and maturation into tumor-specific effector immune cells. The effector cells are increasingly recruited into the imiquimod-modulated tumor site [17,24,25] where they trigger an enhanced tumor-specific immune response which is further maintained by the continuing application of imiquimod. Thus, from tissue findings during immunocryosurgery of BCC [12], we know that the density of CD3^+^ T cells in the tumor tissue had significantly already increased two days after the cryosurgery session. At the same time, the initial, during the early, precryosurgical phase of imiquimod treatment, increased infiltration of the tumor by Treg (Foxp3^+^) cells is reversed. Conclusively, the net effect of cryosurgery during imiquimod treatment of BCC is an abrupt increase in the CD3^+^/Foxp3^+^ ratio at the tumor site. This finding suggests that the tissue perturbations created by the cryosurgery session may probably play a decisive modulating role in the cellular composition of the inflammatory infiltrate during immunocryosurgery, eventually inducing an effective tumor-destructing immune response. Analogous synergy of imiquimod and cryosurgery has also been described at a preclinical level against tumors inexperimental mice, i.e., B16-OVA melanoma [13].

In conclusion, immunocryosurgery seems to set in to action an in situ mechanism that resembles an antitumor vaccination.

## 3. Protocol of Immunocryosurgery

**Figure 2:**
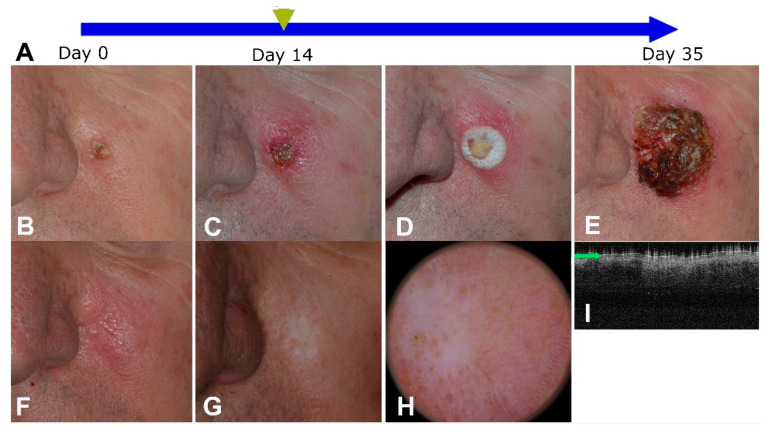
A basal cell carcinoma with a 1-cm maximal diameter at the cheek of a male patient. (**A**): Graphical representation of the treatment process. Imiquimod cream is applied daily for 5 weeks on the tumor area and the cryosurgery session is performed at day 14. (**B**): Tumor at the baseline. (**C**): The tumor area at the cryosurgery day. (**D**): The tumor and a 0.5 cm ring around it are frozen and kept in this state for 15 s. (**E**): Day 35 of the cycle, end of treatment. (**F**): The tumor at 1 month follow-up. (**G**–**I**). The tumor area at 28 months follow-up. (**G**). A flat scar with no signs of tumor relapse and partial repigmentation. (**H**). Corresponding dermoscopic finding. (**I**). Optical coherence tomography of the treated area (NITID, Dermalumics, Spain) displaying homogenous uninterrupted epidermis with normally distributed sebaceous glands. (Figure adapted from Gaitanis et al., Hellenic Derm.-Venereol. Rev. 2018).

Immunocryosurgery is applied in typical 5-week cycles (“a standard 5-week immunocryosurgery cycle”). The right timing and scheduling of each treatment modality is essential to optimize the anti-neoplastic effect. The imiquimod cream (the 5% commercially available product has been used) is applied every night as a thin layer on the lesion and a 0.5 cm rim of surrounding skin. The cryosurgery session is performed on day 14. It consists of two freeze-thaw cycles. Each of them constitutes of 10–20 s of active freezing of the tumor and a 0.5–1.0 cm-rim around it (Figure 2). Given the fact that imiquimod can induce an inflammatory response that involves the surrounding sun-damaged skin and thus blur the borders of the tumor, to trace the tumor localization with certainty, we strongly suggest the use of clinical photographs at baseline which can be used as a reference at the planning of cryosurgery. The ‘active freezing time’ is measured from the moment the target area is completely frozen, i.e., a steady white color has been achieved (Figure 1D). The target area is kept in a frozen state with continuous (large target areas) or intermittent (smaller target areas) bursts of N_2_ from the Cryogun. The latter is adapted with a large tip (A or B size) in order to ascertain rapid freezing. The second freeze-thaw cycle of the session is similarly performed as the first after complete thawing of the lesion. According to our experience, monitoring tissue temperature or thaw duration times do not really impact treatment outcome. If required, e.g., for larger BCC, treatment cycles can be repeated as indicated, or sometimes the duration of the treatment can be extended with continuous application of imiquimod for several weeks and repeated sessions of cryosurgery every 2–3 weeks [10,26].

During immunocryosurgery, a strong inflammatory reaction can develop at the treated site. This is usually more prominent in the first week after the cryosurgery session and lasts up to a few days after the end of treatment. Patients can wash the treated area every morning with simple soap and water along with gentle removal of scabs and crusts that build up there. It should be noted that, often at the end of the treatment, a thick, strong, inelastic, steadily adherent crust develops which can then be removed after closed application of an emollient cream for 30–60 min. This crust has been more rarely reported after treatment with imiquimod monotherapy and has been eloquently termed “arbor cutis” [27].

Eligible for treatment are all patients with a BCC or Bowen’s diseases that can follow instructions and can apply imiquimod for the defined period of time. For the other diagnoses (Kaposi sarcoma, Merkel cell carcinoma, *lentigo maligna*, keratoacanthoma), immunocryosurgery can be used as an adjuvant or alternative to standard therapeutic modalities, such as surgery. The contraindications of immunocryosurgery do not differ from those applied for each of the two combined modalities, i.e., imiquimod and cryosurgery. It should be stressed that treatment of tumors in patients under iatrogenic immunosuppression, including solid organ transplant recipients, is not a contraindication.

The principal adverse event during immunocryosurgery is the already-mentioned local inflammatory response. However, this is an integral part of the action mechanism and its intensity is also a key predictor that goes in parallel to the treatment outcome. However, sometimes this inflammatory response is exuberant and can involve neighboring or relatively distant anatomical sites, especially when these are covered with actinic keratoses. Headache can develop when the tumor is located on the forehead, parietal area and ears, while treated lesions on the periorbital area are associated with eyelid oedema [28,29]. These adverse events are more common after the cryosurgery session and gradually subside towards the end of the treatment.

Patients may also report flu-like symptoms accompanied with tiredness and rigors, particularly in the first week after the cryosurgery session. These physical symptoms have been peaked up in the results of a relevant quality of life study [30]. By reasoning from corresponding pre-clinical and clinical data on liver cryosurgery, we would like to suppose that these symptoms are probably connected to the production and systemic release of pro-inflammatory cytokines by the imiquimod-modified cryoablated tissue. This is also directly supported by our recent findings of a pro-inflammatory cytokines milieu in the serum of patients with BCC treated with immunocryosurgery [11]. However, with unrestricted access to relevant therapy information and to the treating physicians for reassurance, the need for “medical support” during the whole 5-week treatment is reduced and ascertains patient compliance.

Delayed side-effects at the treatment site include the hyper-, or more usually, the hypopigmentation of the area, which slowly improves. A shallow scar that corresponds to the treated tumor (“tumor ghost”) is often observed after a successful treatment. Nevertheless, the aesthetic outcome of the treatment is judged as “good” or “excellent” by doctors and patients (own unpublished data). Interestingly, some patients report a tingling sensation on the treated area after more than a year, which however does not interfere or disturb their daily activities [4,31].

The main advantages of immunocryosurgery from the health system perspective include the low implementation cost, the easy applicability at an outpatient level [31,32], and the minimal training needed for the treating doctors. From the patient perspective, the main advantages result from the combination of a high tumor-controlling efficacy and an excellent aesthetic and functional outcome. This is more obvious in the case of large BCC [33] or lesions close to important anatomical structures (periorbital lesions, nose) for which complex surgical interventions are avoided [28,29]. Immunocryosurgery can be applied to elderly patients with unavoidable co-morbidities without the need to interrupt or adapt anticoagulation therapy. Regarding younger patients, and in contrast to other non-surgical treatments, such as radiation therapy, immunocryosurgery has not been linked to enhanced carcinogenesis risk. Likewise, no reactivation of herpes infection has been observed.

As already mentioned, the main disadvantage of immunocryosurgery is the topical discomfort caused by the induction of the inflammatory response in the treated area. The patients should be encouraged to continue treatment and be reassured for the temporary nature of the irritation. It is usually helpful to demonstrate series of typical cases (before, during treatment, after treatment) [4] in order to ensure compliance.

Subsequently, we will comment on two issues that commonly arise during treatment and are important to strengthen the doctor–patient trust and to achieve the mandatory compliance to the therapy.

### 3.1. The Right “Intensity” of Inflammation

**Figure 3:**
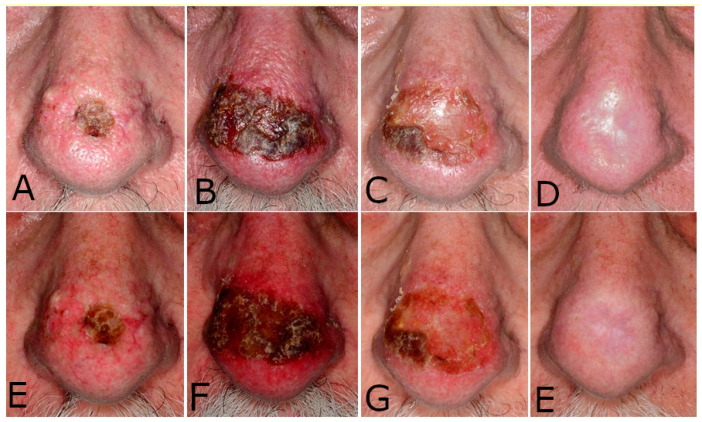
A basal cell carcinoma with indistinct borders in a severe photodamaged nose. The clinical diameter was measured to 18 mm. (**A**–**D**): Parallel polarized photography that highlight the surface of the tumor. (**E**,**F**): Cross-polarized photography that intensifies the subepidermal blood vessels and the erythema. (**A**,**E**): The tumor at baseline. (**B**,**F**): The tumor at day 14, with intense inflammation expanding beyond the tumor borders. The area corresponding to the developed scabs was treated with two cycles of liquid N_2_, 15 s each. (**C**,**G**): Tumor area at the end of treatment. (**D**,**H**): Tumor area at 6 months follow-up. The scar corresponds to the irregular shape of the initial tumor.

In almost all cases an intense inflammatory response develops after the cryosurgery session, even in those patients that after 14 days of imiquimod application presented with minimal inflammation at the tumor site. This is considered a positive predictive sign of therapy outcome, yet there are no means to predict the intensity of this reaction before treatment as it varies significantly between patients but also between lesions within the same patient. Generally, it is more pronounced in older patients with many concurrent actinic keratoses and photodamage (Figure 3). However, the intensity of this inflammatory response, which is directly associated with the discomfort caused, should not lead to the discontinuation of therapy. As a rule of thumb, and in order to avoid an outspreaded application of imiquimod, we instruct the patients to use a third of the commercially available 5% imiquimod sachet (about 4 mg of active ingredient) for BCC up to a 1-cm diameter, a half of the sachet (about 6 mg) for tumors up to a 2-cm diameter, and the whole sachet (about 12 mg) for tumors with a 2–3-cm diameter or larger in maximal diameter.

Absence of inflammation is an exception to the rule and can be observed in a few cases, particularly in patients with underlying hematological malignancies or under immunosuppresive treatment. In these cases, the desired inflammatory response is achieved with the addition of a topical retinoid (tazarotene or tretinoin) every morning on the treated area.

### 3.2. Management of Adverse Events

In contrast to surgery where compliance during the treatment procedure is not an issue, the application of imiquimod is totally dependent on the patient and therefore detailing the procedure and the anticipated adverse events is of paramount importance. We have observed that patients that fail to apply imiquimod the previous 1–2 days prior to the cryosurgery session have a reduced clearance rate.

After the cryosurgery session, it is not uncommon that the patients, especially those with large lesions treatments, may present with some flu-like symptoms including low-grade fever, anorexia, and tiredness. The patients can be reassured that most symptoms commonly subside 7–10 days after the cryosurgery session or certainly within a week after the end of treatment. Again, demonstrating pictures of the treatment procedure is also reassuring to the patient. Availability of the treating physician for a short telephone call or a visit is also important. It should be stressed that, although during immunocryosurgery some “grotesque” clinical pictures of the treated area may develop (Figure 1 and Figure 2), these are completely reversible after the end of treatment and the treating physician can reassure the patient on this.

## 4. Indications of Immunocryosurgery

Immunocryosurgery is a relatively new ablative addition to the available skin cancer treatments, and the spectrum of indications is still under investigation. Principally, it has been used in the treatment of keratinocytic skin cancers, with more experience focusing on the treatment of BCC. Currently, no studies exist that compare directly immunocryosurgery with the standard of care which is surgery. Published data on the use of immunocryosurgery from us or other authors are summarized in Table 1. In the following paragraphs, these indications and a reference to tips of use will be detailed.

### 4.1. Keratinocytic Skin Cancers

The selection of the suitable non-surgical, ablative treatment of keratinocytic skin cancers is determined by the histological diagnosis as well as the general physical condition of the patient, the co-morbidities, and the concurrent medications. These general rules also apply for immunocryosurgery. In general, immunocryosurgery is indicated for the treatment of BCC, Bowen’s disease, and as field treatment of actinic keratoses. Under certain circumstances, it could be considered as a treatment alternative for actinic cheilitis, keratoacanthoma, and low-grade SCC.

#### 4.1.1. Actinic Keratoses, Actinic Cheilitis, in situ Squamous Cell Carcinoma (Bowen’s Disease) and Keratoacanthoma

The efficacy of immunocryosurgery in these epithelial cancers and precancers is highly dependent on the stage of the tumor and its inherent biological behavior. In principle, immunocryosurgery is highly effective and thus indicated as a treatment of in situ (actinic keratoses, actinic cheilitis, in situ squamous cell carcinoma) or low grade (keratoacanthoma) cutaneous neoplasms. However, this cannot be expanded to include cSCC as it has unpredictable behavior which will be further explained in a following section.

##### Actinic Keratoses

In the treatment of actinic keratoses, immunocryosurgery combines the strong therapeutic potential of a spatially targeted treatment (cryosurgery) and a field therapy (imiquimod). Thus, it is suitable for treatment of large anatomical areas (up to 25 cm^2^) or multiple confluent lesions. However, application of imiquimod in an extended large field of photodamaged skin (i.e., >25 cm^2^ of skin of individual or confluent lesions) can induce extreme inflammation beyond the clinical margins of the lesions [43]. We have accordingly adapted the protocol to maintain an equilibrium between inflammatory irritation and the efficacy of the treatment. The patient applies imiquimod in the target lesions, not daily as in the ‘classic cycle’ but every other day for 14 days, including the night before the cryosurgery session. The cryosurgery session for actinic keratoses consists of two freeze-thaw cycles with liquid N_2_, of only 10 s each. The patient applies the imiquimod cream the same day of the cryosurgery session and every other night for four subsequent applications. In total, imiquimod is applied for 11 nights, and, to assist the patient, a detailed application calendar can be given. For lesions with a retarded response, this cycle can be repeated and in the absence of inflammation a topical retinoid can be added. This approach can be used also for the treatment of hypertrophic AK.

##### Actinic Cheilitis (Solar Cheilosis)

Actinic cheilitis can progress to highly invasive squamous cell carcinoma of the lower lip [44]; for this reason, we have evaluated the typical 5-week immunocryosurgery protocol for this condition in eight patients [45]. Although the efficacy was satisfactory and solar cheilosis improved to all patients, as measured with automatic image analysis, a significant impact on the quality of life after the cryosurgery session resulted in most cases, including the inability to eat solid food for a few days. Therefore, this approach is suitable only for highly motivated patients after being informed in detail about the process.

##### In Situ Squamous Cell Carcinoma (Bowen’s Disease)

Both cryosurgery and imiquimod are considered as moderately effective modalities for Bowen’s disease [46]. However, with a typical 5-week immunocryosurgery cycle, an excellent and sustainable result is achieved [6,38]. Bowen’s disease can sometimes acquire extensive size or can be difficult to treat in anatomic locations, as are the fingers [39]. In these cases, immunocryosurgery is an excellent therapeutic solution. Small lesions can be effectively eradicated with the typical 5-week treatment cycle while larger lesions, especially those located on the head, can be treated in subsections of 4–5 cm^2^. In the case of small recurrences (diameter up to 0.5 cm) within a large treated area, cryosurgery monotherapy can suffice while, for larger lesions, a repeat immunocryosurgery cycle will be usually needed. It should be stressed that, up to now, and in contrast to observations reported for imiquimod monotherapy [47], we have not observed evolution of Bowen’s disease to invasive cSCC after immunocryosurgery in 30 patients (36 lesions) for a period of time ranging 6–24 months (Table 1) [6,38].

##### Keratoacanthoma

Keratoacanthoma diagnosis entails a certain degree of uncertainty as it cannot always be safely differentiated from cSCC [48,49] and, therefore, surgical excision is recommended as the first line of treatment [49]. However, recent advances in the understanding of the pathophysiology of this tumor [50,51] provide us with the prerequisite tools for the effective confirmation of the diagnosis of keratoacanthoma and have helped in the therapeutic assessment of this self-regressing tumor.

Regarding immunocryosurgery, we have effectively used this approach in combination with intralesional injections of methotrexate as an alternative to the treatment of keratoacanthoma [7]. After taking a confirmatory biopsy of the newly diagnosed keratoacanthoma at baseline, we apply a relatively mild cryosurgery session (liquid N_2_, open spray, 2 cycles of 15 s each) followed by 5 mg of intralesional methotrexate immediately afterwards. In the following 2 weeks, the patient applies imiquimod every night on the lesion. The cryosurgery session is repeated in two-weekly intervals until complete regression of the tumor.

By transferring our experience from large BCC [26,33] (including not published data) and cSCC cases [10], we know that the immunocryosurgery can be potentiated with repeated sessions (every 2–3 weeks) of cryosurgery while the tumor is under continuous topical imiquimod treatment.

#### 4.1.2. Cutaneous Squamous Cell Carcinoma

**Figure 4:**
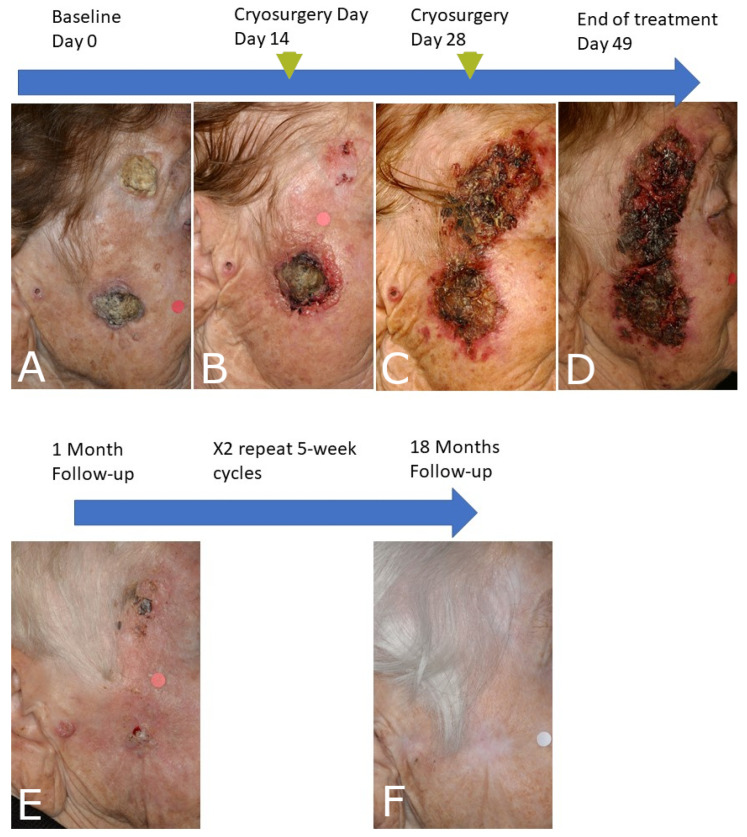
(**A**). Squamous cell carcinoma (diameter 3 cm) and a cutaneous horn arising within a carcinogenesis field of actinic keratoses. A squamous carcinoma progressing into this extended actinic keratosis field is also evident in the preauricular area. (**B**–**D**): The patient was treated with a ‘prolonged’ 7-week daily imiquimod and two cryosurgery sessions (liquid N_2_, 2 cycles, 30 s each) at weeks 2 and 4 of the immunocryosurgery cycle. (**B**,**C**). The tumor remnants one month after the end of treatment and the preauricular lesion (**E**) were treated with two additional, standard 5-week immunocryosurgery cycles, demonstrating sustained clearance at 18 months follow-up (**F**).

It should be emphasized that the response of cSCC to immunocryosurgery is unpredictable. In patients with large, advanced cSCC in whom adapted intense immunocryosurgery schemes were applied as a palliative modality, these initially responded but subsequently relapsed and had to be treated with another approach. Notably, relapses, or the emergence of metastasis, have been reported with imiquimod monotherapy [52], although isolated reports describe effective treatment of cSCC, even with imiquimod monotherapy [53,54,55,56,57]. Immunocryosurgery is more effective in the case of relatively stable cSCC, i.e., tumors that have not grown more than 20% the last 12 months, in elderly patients and under close clinical follow up it can be recommended in order to treat timely recognized probable relapses or delayed responses (Figure 4). With this approach we have achieved long standing complete remission in certain cases of cSCC (>12 months; eight tumors in four patients) with neo-adjuvant use of intralesional methotrexate (Table 1) [10].

#### 4.1.3. Basal Cell Carcinoma

Most published data as well as unpublished observations pertain to the application of immunocryosurgery for the treatment of BCC. For this reason, we will report our experience on the treatment of locally confined BCC (tumors with clinical diameter ≤2 cm), locally advanced BCC (tumors with clinical diameter 2–5 cm), as well as multiple concurrent or metachronous BCC.

##### Locally confined BCC

To date, the larger part of the experience in the treatment of BCC with immunocryosurgery entails tumors with a maximal diameter up to 2 cm, irrespective of anatomical localization, histologic diagnosis, both primary and relapses after surgery. These cases represent 80–90% of all BCC [58,59] and the efficacy of one typical 5-week cycle of immunocryosurgery reaches 97.5% [4]. As detailed previously in this text, the 5-week treatment cycle corresponds to daily imiquimod application for 5 weeks and a session of cryosurgery at week 2 of the cycle (2 cycles of 15–20 s each). Follow-up is scheduled at 1 month after the last application of imiquimod, at 3 months, 6 months for the first 2 years, and yearly afterwards.

Regarding the efficacy of the modality, it is comparable to the first-line surgical therapy. Notably, also in cases of no-response or relapse after one cycle of immunocryosurgery with a second standard 5-week cycle, a 99% clearance rate of BCC with a maximal diameter of ≤2 cm is achieved [4]. This result was maintained, as 97% of tumor sites remained relapse-free up to 5 years after immunocryosurgery [5]. This outcome is comparable to optimally performed surgical methods for this particular neoplasm [60].

It should be stressed that the efficacy of immunocryosurgery is significantly higher in comparison to imiquimod monotherapy for nodular BCC or to that of cryosurgery alone, particularly for the employed moderate freezing times <20 s. Notably, tumors that are indicated for Mohs surgery, such as the ones with undefined borders or in localizations prone to relapse after surgery, do not constitute a contraindication to immunocryosurgery.

In the recent European Guidelines for the diagnosis and treatment of BCC, immunocryosurgery is recommended as one of the alternative treatments for tumors that are not anticipated to have a satisfactory outcome with surgical excision [61]. According to our experience, we suggest that immunocryosurgery can be used as a first-line alternative to surgical excision for the treatment of BCC. Typical examples where immunocryosurgery is not suitable, is BCC localization on the back, where the patients cannot apply imiquimod by themselves or younger patients that have constant contact with people and want to avoid explaining the inflammation in their face.

##### Locally Advanced Basal Cell Carcinoma

This corresponds to tumors with a maximal diameter of >2 cm, in which the efficacy of a standard 5-week immunocryosurgery cycle is restricted as the tumor size increases. For tumors 3–5 cm in maximal diameter, the efficacy is confined to approximately 75%through our own empirical observations. For these tumors, we apply longer cryosurgery sessions (2 × 20 s) and the tumor is frozen in 1–2 cm^2^ overlapping sectors, sometimes under local anesthesia. During follow-up, diagnostic biopsies are performed and, if necessary, immunocryosurgery can be repeated.

Currently, we are also evaluating longer treatment cycles [33] or the addition of adjuvants during the cryosurgery session, such as the anti-vascular endothelial growth factor monoclonal antibody bevacizumab [26].

##### Multiple Locally Confined Basal Cell Carcinoma

BCC can appear as multiple tumors in patients that have been subjected to carcinogenic and/or immunosuppressive factors (e.g., ultraviolet radiation, ionizing radiation, transplantation of solid organs, etc.). These tumors are prone to confluency which threatens vital anatomical structures or constitute surgical fields of many centimeters where surgical treatment or therapeutic radiation would constitute a challenge as it would leave significant anatomical or functional residua on the treated area.

Immunocryosurgery ascertains the maximal preservations of tissues and constitutes an ideal treatment selection for these patients. At this point, a special note pertaining to patients with Gorlin–Goltz syndrome is required. Although we did not have the chance to treat a patient with this diagnosis, taking into consideration the data from the independent efficacy of imiquimod and cryosurgery [62,63], we strongly believe that immunocryosurgery should also be efficacious in the BCC that appear in these patients.

##### Palliative Therapy for Locally Advanced Basal Cell Carcinoma

For some patients with locally advanced BCC, palliative therapy is a more realistic approach in comparison to cure. This applies for neglected cases of BCC which are not amenable to conventional therapy as they have progressed to aggressive tumors with potential of unlimited local extension. In these cases, immunocryosurgery can be used as a palliative intervention with the aim to reduce the tumor load and protect significant anatomical structures. It would be interesting to enquire combinations of immunocryosurgery with systematic therapies for locally advanced BCC, such as the hedgehog inhibitors (e.g., vismodegib) or the recent addition of the immune stimulating anti-PD antibodies [61]. A combination of two immune stimulating therapies may mutually enhance their systemic and local effectiveness.

### 4.2. Non Keratinocytic Skin Cancers

#### 4.2.1. *Lentigo Maligna* and Malignant Melanoma

*Lentigo maligna* is an in situ melanoma that has the potential to proceed to an invasive melanoma in 5% of the patients during their lifetime. Both monomodal imiquimod and cryosurgery have been used in the treatment of *lentigo maligna* [64,65] as well as immunocryosurgery. Notably, the first case report of the clinical use of immunocryosurgery was a case of *lentigo maligna* [1]. Additional cases with successful eradication of this in situ melanoma have followed [9], including a case of periocular *lentigo maligna* [40]. As with immunocryosurgery, for keratinocytic skin cancers, an adequate amount of inflammatory response is typical for the successful eradication of *lentigo maligna*; this can be achieved with repeated cryosurgery sessions during continuing imiquimod application, as required [41]. Close follow-up and targeted biopsies are needed after the end of treatment in order to confirm the absence of tumor rests.

To date, immunocryosurgery is not recommended for the treatment of malignant melanoma. However, there are two instances in which it could be used as an alternative or adjuvant treatment: (i) After a diagnostic excision biopsy in order to treat possible remnants of the tumor in anatomical areas where a wider excision according to the guidelines could compromise vital structures. In contrast, in immunocryosurgery the inflammation induced is temporary and the therapy only targets the involved by the tumor area, as the experience from the treatment of BCC shows. (ii) As palliative therapy for cutaneous melanoma metastases. In the latter case, the induction and maintenance of a considerable inflammatory response is of paramount importance; for this reason, extended treatment schemes with repeated cryosurgery sessions and/or adjuvant topical retinoids could be used until clinical regression is achieved [66,67].

#### 4.2.2. Sarcoma Kaposi

**Figure 5:**
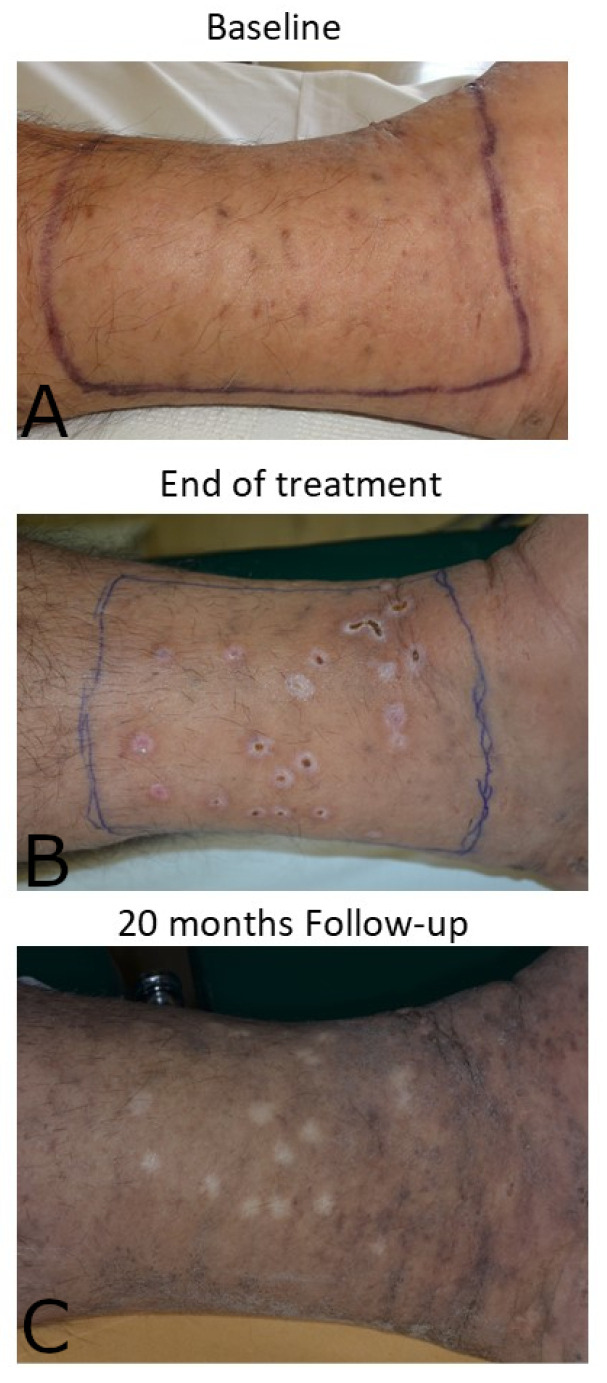
Inner malleolus of an adult patient treated for classical Kaposi sarcoma with interferon-α. (**A**). A group of lesions treated concurrently to subcutaneous interferon-α with a standard 5-week immunocryosurgery cycle at baseline; (**B**). At the end of the treatment cycle; (**C**). At the 20-month follow-up.

Skin lesions of classic sarcoma Kaposi respond promptly to immunocryosurgery. Until now, we have applied this approach to elderly patients that were concurrently treated with interferon-α (Figure 5). The lesions are treated in groups of 5–10 that sum up an up to 25 cm^2^ area each time. The patient starts to apply imiquimod in other lesions at the end of a 5-week treatment cycle. Interestingly, the induced inflammatory response is not that intense in comparison with the treatment of keratinocytic skin cancers, although the efficacy is satisfactory when we compare the treated with the untreated areas.

#### 4.2.3. Merkel Cell Carcinoma

Merkel cell carcinoma is a rare aggressive malignant tumor which appears in elderly patients and is characterized by local relapses after surgical removal of the primary tumor. We have described the treatment of in transit lesions appearing in a patient 6 months after the removal of the primary carcinoma [8]. In particular, more than 15 cutaneous-subcutaneous lesions were effectively treated with standard 5-week immunocryosurgery cycles. It is of particular interest that a concurrent paraneoplastic arthritis also subsided after the treatment of metastasis.

### 4.3. Non-Malignant Skin Lesions

#### 4.3.1. Cutaneous and Genital Warts

Imiquimod is licensed for the treatment of genital warts and cryotherapy [68] and is a widely employed treatment alternative of this common genital infection. The compliance and thus the effectiveness of the combined treatment in the form of immunocryosurgery can be restricted by the local intense inflammation. Therefore, for small condylomata, we usually employ the alternate day scheme described for the treatment of AK. In case of strong inflammation, the quantity of the applied imiquimod can be adequately reduced.

For large, outgrowing lesions, we recommend the standard daily scheme and cryosurgery sessions every two weeks according to the clinical response (flattening) of the lesions.

Plantar warts can prove therapy-resistant, especially in subpopulations of immunocompromised individuals. The alternate day immunocryosurgery scheme with cryosurgery every two weeks could be used as an efficacious alternative for resistant warts. As a rule, minimal inflammation is observed with this approach; in case of failing or delayed response, the frequency of imiquimod application can be increased to a daily scheme for non-responders.

#### 4.3.2. Pyogenic Granuloma

Imiquimod and cryosurgery as monotherapies are used for the treatment of pyogenic granuloma [69,70]. We have observed that their combination is effective when imiquimod is used daily and cryosurgery (liquid N_2_, 2 cycles, 10 s each) is applied every two weeks guided by the clinical response. Excellent healing without scaring is achieved, and, interestingly, the treatment of these lesions is not associated with distinct inflammation.

It should be stressed that the diagnosis of pyogenic granuloma should be confirmed in order to avoid the delay of treatment of an amelanotic melanoma [71].

## 5. Conclusions

In conclusion, the combination of imiquimod and cryosurgery within the frame of immunocryosurgery is a potent and efficacious treatment for keratinocytic skin cancers that enables a lasting therapeutic result combined with maximal healthy tissue preservation. BCC remains the main domain of immunocryosurgery application. However, the spectrum of diagnoses that are amenable to treatment with immunocryosurgery is currently actively explored, as well as the impact of possible adjuvants.

## Figures and Tables

**Table 1:** Published studies on the application imiquimod and cryosurgery for the treatment of lentigo maligna and keratinocytic skin cancers.

Tumor	Type of Study	Number of Patients/ Tumors	Concluded Therapy	Efficacy (Clearance %)	Follow Up (Months)	Reference
Basal cell carcinoma	Retrospective, observational cohort study	41	41	N/A		[34]
Basal cell carcinoma	Retrospective, case series	13/21	13	20/21 (95%)	18–24	[35]
Basal cell carcinoma	Prospective, randomized, open-label, two arm study comparing 5 weeks daily imiquimod with cryosurgery at Day 0 (“cryoimmunosurgery”) vs. Day 14 (“immunocryosurgery”) (NCT01212549)	14/17	12/14		Study interrupted at interim analysis	[3]
Basal cell carcinoma	Extended/repeated immunocryosurgery cycles with bevacizumab as an add-in adjuvant for large (>2 cm diameter), multifocal relapses after surgery, relapses after standard immunocryosurgery	7/7	7	3/7 (43%)	18–48	[26]
Basal cell carcinoma	Phase III, prospective, interventional, single-arm evaluating a 5-week cycle of immunocryosurgery in non-superficial BCC with diameter ≤2 cm (NCT01212562)	83/124	79	With repeat immunocryosurgery cycles effectiveness reached 97.1 ± 1.6% per protocol or 93.2 ± 2.3% per intention to treat analysis	>5 years	[4,5]
Basal cell carcinoma		24/36	24	35/36 (97.2%) tumor sites, 5 tumors required repeat cryosurgery/immunocryosurgery at one month follow-up	3–24	[31]
Basal cell carcinoma (periocular)	Case-series	16/16	16	13/16 (81%)	3–60	[28,29]
Basal cell carcinoma (periocular)	Case	1	1	Clearance	12	[36]
Basal cell carcinoma	Case of neglected BCC	1	1	1/1	24	[33]
Basal cell carcinoma	Cases	2/17	2	Clearance	18–24	[37]
Bowen’s disease	Case series	8/11	8	11 (100%)	6–24	[38]
Bowen’s disease	Case series	21/24	21	22/24 (91.7%)	6–60	[6]
Bowen’s disease	Case	1/1	1	1/1	12	[39]
Squamous cell carcinoma	Case series	4/8	4	6/8 (75%)	12	[10]
*Lentigo Maligna*	Case	1/1	1	1/1	26	[1]
*Lentigo Maligna*	Cases	2/2	2	1/2 (50%)	15–18	[40]
*Lentigo Maligna*	Cases	3/3	3	3/3 (100%)	41–48	[9]
*Lentigo Maligna*	Case	1/1	1	1/1	48	[41]
*Lentigo Maligna*	Case	1/1	1	1/1	21	[42]

## Data Availability

Not applicable.
